# Draft Genome Sequence of the Polyhydroxyalkanoate-Producing Bacterium *Burkholderia sacchari* LMG 19450 Isolated from Brazilian Sugarcane Plantation Soil

**DOI:** 10.1128/genomeA.00313-15

**Published:** 2015-05-07

**Authors:** Paulo Moises Raduan Alexandrino, Thatiane Teixeira Mendonça, Linda Priscila Guamán Bautista, Juliano Cherix, Gabriela Cazonato Lozano-Sakalauskas, André Fujita, Edmar Ramos Filho, Paul Long, Gabriel Padilla, Marilda Keico Taciro, José Gregório Cabrera Gomez, Luiziana Ferreira Silva

**Affiliations:** a Department of Computer Science, Institute of Mathematics and Statistics, University of São Paulo, São Paulo, Brazil; b Institute of Biomedical Sciences, University of São Paulo, São Paulo, Brazil; c Institute of Pharmaceutical Science, King’s College London, London, United Kingdom; d Faculty of Pharmaceutical Sciences, University of São Paulo, São Paulo, Brazil

## Abstract

*Burkholderia sacchari* LMG 19450, isolated from the soil of a sugarcane plantation in Brazil, accumulates large amounts of polyhydroxyalkanoates from sucrose, xylose, other carbohydrates, and organic acids. We present the draft genome sequence of this industrially relevant bacterium, which is 7.2 Mb in size and has a G+C content of 64%.

## GENOME ANNOUNCEMENT

*Burkholderia sacchari* LMG 19450 was isolated in the 1990s from the soil of a sugarcane plantation in Brazil (1) and was described as a new species (2). This bacterium has attracted interest from industry because of its capability to metabolize different carbon sources (sucrose, xylose, organic acids, etc.), reach high cell densities, and accumulate high levels of polyhydroxyalkanoates (PHA) (3–5) and also because it is sensitive to a large number of antibiotics (3). The aim of sequencing the genome was to identify genes involved in the catabolism of xylose and other sugars derived from biomass, as well as genes involved in PHA metabolism.

Genomic DNA was extracted using the DNeasy blood and tissue kit (Qiagen). The DNA was concentrated to 353.9 ng/µL, and the quality was assessed by agarose gel electrophoresis and on a NanoDrop spectrophotometer (Thermo Scientific). Whole-genome sequencing was performed by Macrogen (Seoul, South Korea) using the 454 GS FLX sequencing platform, which generated 785,669 reads. The sequencing reads were assembled with Newbler (Roche), and contigs annotation was carried out using the Rapid Annotation Server Subsystem Technology (RAST) (6).

The draft genome is composed of 121 contigs with a total length of 7,265,069 bp (depth of coverage, 49×) and a G+C content of 64.03%. The mean size of the contigs is 60,042 bp, and the *N*_50_ is 208,943 bp. RAST identified 6,741 coding regions, among which there were genes related to carbohydrate catabolism, e.g., xylose transporter ATP-binding subunit (*xylG*), xylose transporter substrate-binding protein (*xylF*), xylose isomerase (*xylA*), xylulokinase (*xylB*), and xylose operon regulatory protein (*xylR*) for xylose catabolism; to fatty acid catabolism, e.g., acyl-CoA synthetase (*fadD*), acyl-coenzyme A (CoA) dehydrogenase (*fadE*), enoil-CoA hydratase-*S*-specific (*fadB*-*fadJ*); and to PHA metabolism, e.g., polyhydroxyalkanoic acid synthase (*phaC*) and 3-ketoacyl-CoA thiolase (*phaA*). No pathogenesis-related gene was found in B. sacchari LMG 19450. Only one gene associated with resistance to antibiotics, encoding undecaprenyl diphosphatase, was found, and it has been associated with resistance to bacitracin (7).

Considering that this bacterium has the potential to convert a wide range of carbon sources, *B. sacchari* LMG 19450 represents a promising candidate for the production of PHA and other biobased products.

### Nucleotide sequence accession numbers. 

This whole-genome shotgun project has been deposited at DDBJ/EMBL/GenBank under the accession number JTDB00000000. The version described in this paper is the first version, JTDB01000000.

## References

[1] Gomez JGC, Rodrigues MFA, Alli RCP, Torres BB, Netto CLB, Oliveira MS, da Silva LF 1996 Evaluation of soil Gram-negative bacteria yielding polyhydroxyalkanoic acids from carbohydrates and propionic acid. Appl Microbiol Biotechnol 45:785–791. doi:10.1007/s002530050763.

[2] Brämer CO, Vandamme P, da Silva LF, Gomez JG, Steinbüchel A 2001 Polyhydroxyalkanoate-accumulating bacterium isolated from soil of a sugar-cane plantation in Brazil. Int J Syst Evol Microbiol 51:1709–1713. doi:10.1099/00207713-51-5-1709. 11594600

[3] Silva LF, Gomez JGC, Oliveira MS, Torres BB 2000 Propionic acid metabolism and poly-3-hydroxybutyrate-*co*-3-hydroxyvalerate, (p 3HB–*co*-3HV) production by *Burkholderia* sp. J Biotechnol 76:165–174. doi:10.1016/S0168-1656(99)00184-4. 10656331

[4] Silva LF, Taciro MK, Michelin Ramos ME, Carter JM, Pradella JG, Gomez JG 2004 Poly-3-hydroxybutyrate (P3HB) production by bacteria from xylose, glucose and sugarcane bagasse hydrolysate. J Ind Microbiol Biotechnol 31:245–254. doi:10.1007/s10295-004-0136-7. 15221664

[5] Mendonça TT, Gomez JG, Buffoni E, Sánchez Rodriguez RJ, Schripsema J, Lopes MS, Silva LF 2014 Exploring the potential of *Burkholderia sacchari* to produce polyhydroxyalkanoates. J Appl Microbiol 116:815–829. doi:10.1111/jam.12406. 24279348

[6] Aziz RK, Bartels D, Best AA, DeJongh M, Disz T, Edwards RA, Formsma K, Gerdes S, Glass EM, Kubal M, Meyer F, Olsen GJ, Olson R, Osterman AL, Overbeek RA, McNeil LK, Paarmann D, Paczian T, Parrello B, Pusch GD, Reich C, Stevens R, Vassieva O, Vonstein V, Wilke A, Zagnitko O 2008 The RAST Server: Rapid Annotations using Subsystems Technology. BMC Genomics 9:75. doi:10.1186/1471-2164-9-75. 18261238 PMC2265698

[7] El Ghachi M, Derbise A, Bouhss A, Mengin-Lecreulx D 2005 Identification of multiple genes encoding membrane proteins with undecaprenyl pyrophosphate phosphatase (UppP) activity in *Escherichia coli*. J Biol Chem 280:18689–18695. doi:10.1074/jbc.M412277200. 15778224

